# Chronic lead poisoning in Iran; a silent disease

**DOI:** 10.1186/2008-2231-20-8

**Published:** 2012-08-28

**Authors:** Omid Mehrpour, Parissa Karrari, Mohammad Abdollahi

**Affiliations:** 1 Medical Toxicology and Drug Abuse Research Center (MTDRC), Birjand University of Medical Sciences, Pasdaran Avenue, Birjand, Iran; 2 Department of Clinical Toxicology and Forensic Medicine, Faculty of Medicine, Birjand University of Medical Sciences (BUMS), Ghaffari Avenue, Birjand, Iran; 3 Department of Toxicology and Pharmacology, Faculty of Pharmacy, and Pharmaceutical Sciences Research Center, Tehran University of Medical Sciences (TUMS), Poursina Avenue, Tehran, 1417614411, Iran

## 

Lead poisoning is one of the oldest, permanent hazards in the world and Iran is not excluded and has the same risk of lead toxicity. Humans have known about the potential hazards of lead poisoning for centuries. Lead is widespread in natural substances, and almost all people are in touch with this insidiously toxic heavy metal in different ways either in workplace or at homes. The level of exposure is higher in most of countries because of extensive use of lead containing materials in normal life and the environment. Published data from Iran show that people in some jobs such as mine workers [1], employees of paint factories [2], workers of copying centers [3], bus drivers [4], and tile making factories [5] are in higher risk of lead toxicity.

The problem would be worse if we consider that even foods have high concentration of lead, for examples vegetables [6], powdered milk [7], bread [8], machine-made lemon juice [9] and tomato paste [10], peanut [11], raw milk [12] and black tea [13,14] as well as many fishes from Caspian sea, Persian gulf, and farmed fishes may have higher levels of lead [15-17]. Studies from Iran have confirmed that source of drinking water such as water wells, cistern in rural area and ground waters of some area contain lead because of many of industrial waste materials leak into underground water resources [18-20]. Besides we should be afraid of leakage of trace metal from corrosion of municipal drinking water distribution in the cities.

Make up products are another source of lead. Traditional eye make ups such as powder of Surma and powders of Kohl, which are used in Iran and other Middle East countries, contain lead and due to the long time contact with skin and eye mucosa they can cause symptoms of lead toxicity in blood and eye [21]. Although in some countries, addition of compounds containing lead and other toxic metals to toys are prohibited but results of some surveys in Iran showed that plastic toys and other PVC products manufactured for children in some area are contaminated with lead [22]. Another concern of lead exposure and lead toxicity is due to addition of lead to opium which may be a concern of opium addicted people [23,24].

Lead sources even available in medical products and drugs, for example some oral herbal drops which is available in markets and Amalgam of tooth restoration are other materials around us that make us in great danger of lead toxicity [25,26].

In another study, it was reported that 40% of randomly selected children had high level of lead in their blood which clearly show importance of screening test for lead poisoning in the population [27].

In general, data from Iran clearly show that everyone is in great danger of exposure and lead toxicity. Because of lead poisoning is a silent disease, screening of this disease have a high priority and with consideration of, we suggest screening test for everyone with nonspecific signs and symptoms, especially in subacute and chronic form like abdominal pain, constipation, irritability and anemia or even asymptomatic patients [28].

Several metal chelators have been used to manage lead toxicity in the case of accidental lead poisoning, but none of them are useful or even cost-benefit [29] in reducing lead burden in chronic lead exposure, so we suggest undertaking further precautions and designing programs in this regard. Of course in serious cases that higher blood lead levels are confirmed, use of effective oral antidote dimercaptosuccinic acid (Succimer) is recommended.

Various countries have started programs and policies to prevent and treat morbidities associated with this toxic heavy metal so in this editorial we want to make attention of readers about dangers of lead poisoning in Iran and to request immediate policies for prevention of serious health problem. Our final message is that screening for blood lead level should be performed for all people who seems are in exposure and risk. This needs portable rapid samplers and lead analyzers in easier samples like saliva [30][,31].

## Authors’ contribution

OM and PK did bibliography and drafted the article. MA gave the idea and completed/edited/revised the article. All authors read and approved the final manuscript.

## Authors’ information

Mohammad Abdollahi is an Adviser to the World Health Organization (WHO) for providing WHO guidelines on the prevention and management of lead poisoning. He is doing his best to integrate identification of lead toxicity in human and its hazards to the environment.
